# Immune characteristics and clinical significance of peripheral blood lymphocytes in breast cancer

**DOI:** 10.1186/s12885-024-11815-8

**Published:** 2024-01-09

**Authors:** Hongyu Gao, Dengjie Ouyang, Xinyu Guan, Jiachi Xu, Qitong Chen, Liyun Zeng, Jian Pang, Qiongyan Zou, Ke Qian, Wenjun Yi

**Affiliations:** 1 Department of General Surgery, The Second Xiangya Hospital, Central South University, No.139 People’s Road, Changsha, Hunan 410011 P.R. China; 2 Clinical Research Center For Breast Disease In Hunan Province, Changsha, 410011 China; 3 Department of General Surgery, Xiangya Hospital Central South University, Changsha, China

**Keywords:** Breast Cancer, Peripheral blood lymphocytes, Nomogram, RFS

## Abstract

**Background:**

In the context of breast cancer (BC), the correlation between lymphocytes and clinical outcomes, along with treatment response, has garnered attention. Despite this, few investigations have delved into the interplay among distinct peripheral blood lymphocyte (PBL) types, immune attributes, and their clinical implications within the BC landscape.

**Methods:**

The primary objective of this study was to scrutinize the baseline status of PBL subsets in patients with primary BC, track their dynamic changes throughout treatment, and ascertain their interrelation with prognosis. Flow cytometry was employed to analyse PBLs from a cohort of 74 BC patients.

**Results:**

Our analysis revealed that baseline levels of Treg and PD-L1 + T cells were lower in BC patients compared to the reference values. Notably, a disparity in baseline PD-L1 + T cell levels surfaced between patients who underwent adjuvant therapy and those subjected to neoadjuvant therapy (NAT). Furthermore, a meticulous evaluation of PBL subsets before and after treatment underscored discernible alterations in 324 + T cells and CD19 + CD32 + B cells over the course of therapy. Strikingly, heightened CD4 + T cell levels at baseline were linked to enhanced event-free survival (EFS) (*p* = 0.02) and a robust response to chemotherapy.

**Conclusions:**

These results indicate that PBLs may serve as a significant marker to assess the immune status of BC patients, and therapy has the potential to modify patient immune profiles. In addition, peripheral blood CD4 + T cell levels may serve as promising biomarkers for diagnosis and prognosis in future studies of BC.

**Supplementary Information:**

The online version contains supplementary material available at 10.1186/s12885-024-11815-8.

## Introduction

Breast cancer (BC) is the most common malignant tumor among women on a global scale, and its incidence is increasing yearly, with the number of new BC patients reaching 2.26 million worldwide in 2020 [1, 2]. BC, previously believed to exhibit low immunogenicity, has been increasingly recognized for its involvement with the immune system, particularly in the case of triple-negative breast cancer (TNBC) [3, 4]. In recent years, immunotherapeutic drugs represented by immune checkpoint inhibitors (ICIs) have carried out a range of clinical studies in the area of BC treatment and achieved encouraging results. For example, PD-1 or PD-L1 blockers in combination with chemotherapy have been shown to improve the prognosis of patients with TNBC [5–7]. Tremelimumab, an FDA-approved CTLA-4 blocker for liver cancer and non-small cell lung cancer, has also demonstrated potential benefits through immune activation in BC patients [8–10]. Moreover, several preclinical trials have demonstrated the positive efficacy of Chimeric antigen receptor T (CAR-T) cell therapy in BC treatment, and multiple ongoing clinical trials are evaluating the use of CAR-T for treating BC [11, 12]. Although immunotherapy shows great promise in BC, it still faces the problems of a limited response rate and tumor resistance [13–17]. Therefore, it is very important to explore the immune status in BC and further identify key regulatory factors.

The correlation between higher levels of tumor infiltrating lymphocytes (TILs) and improved prognosis in BC has been extensively investigated [18–21]. Higher levels of TILs have been shown to be associated with reduced recurrence and improved overall survival (OS) [22]. Increased infiltration of CD8 + T, T helper 1(Th1), T follicular helper (Tfh) and natural killer (NK) cells in tumors is generally considered to be associated with better prognosis [23–25]. In contrast, T helper (Th2) and regulatory T cells (Treg) cells were thought to promote tumor progression and metastasis [26, 27].

Peripheral blood lymphocytes (PBLs) are more accessible, noninvasive, and far less costly than TILs [28]. In addition, several studies indicate that PBLs predicted response to Neoadjuvant therapy (NAT) on some tumors, including prostate cancer, oesophageal cancer, and lung cancer [29–31]. Nevertheless, our understanding of the diagnostic significance of PBL subsets in assessing the immune status of BC patients remains limited. And it still unclear how chemotherapy causes changes in PBL subsets. Therefore, exploring the landscape of PBLs subsets can enhance our comprehension of the intricate interplay between the immune system and diseases, thus yielding valuable insights for precision medicine [32].

Therefore, this study aims to analyse the baseline status of PBL subsets in BC patients and the changes induced by therapy, as well as the association between PBL subsets and prognosis. Indeed, our analysis suggests that PBL subsets levels at baseline are associated with patient prognosis, especially the levels of CD4 + T and CD19 + CD32 + B cells at baseline. Moreover, there may be a certain regularity in the changes of PBL subsets during chemotherapy, which needs to be further explored.

## Methods

### Clinical samples and databases

Peripheral blood (PB) samples were collected from a cohort of 74 patients with BC who received treatment at the Second Xiangya Hospital, Central South University in Changsha, China.

Considering the differences in immunogenicity of different breast cancer subtypes and the impact of different chemotherapy regimens on the immune environment [4, 33–35], patients can be classified as follows: luminal-like (ER or PR positive, or both; HER2 negative, HR + HER2-); HER2 positive (HER2 positive, HER2+); or triple negative (ER negative, PR negative, and HER2 negative, TNBC).

The chemotherapy regimens in this study can be found in Table 1.

**Table 1:** Chemotherapy regimens

Reginmen	Drug types	Amounts	Treatment courses
AC-P/T	Adriamycin	60 mg/m^2^	q3w x 4
	Cyclophosphamide	600 mg/m^2^	q3w x 4
	Paclitaxel	175 mg/m^2^	q3w x 4
	Docetaxel	75 mg/m^2^	q3w x 4
EC-P/T	Epirubicin	100 mg/m^2^	q3w x 4
	Cyclophosphamide	600 mg/m^2^	q3w x 4
	Paclitaxel	175 mg/m^2^	q3w x 4
	Docetaxel	75 mg/m^2^	q3w x 4
TAC/PAC	Docetaxel	80 mg/m^2^	q3w x 6
	Paclitaxel	75 mg/m^2^	q3w x 6
	Adriamycin	50 mg/m^2^	q3w x 6
	Cyclophosphamide	600 mg/m^2^	q3w x 6
TEC/PEC	Docetaxel	80 mg/m^2^	q3w x 6
	Paclitaxel	75 mg/m^2^	q3w x 6
	Epirubicin	50 mg/m^2^	q3w x 6
	Cyclophosphamide	600 mg/m^2^	q3w x 6

*HER2+: In patients with HER2 + BC, anti-HER2 therapy is added

**Fig. 1:**
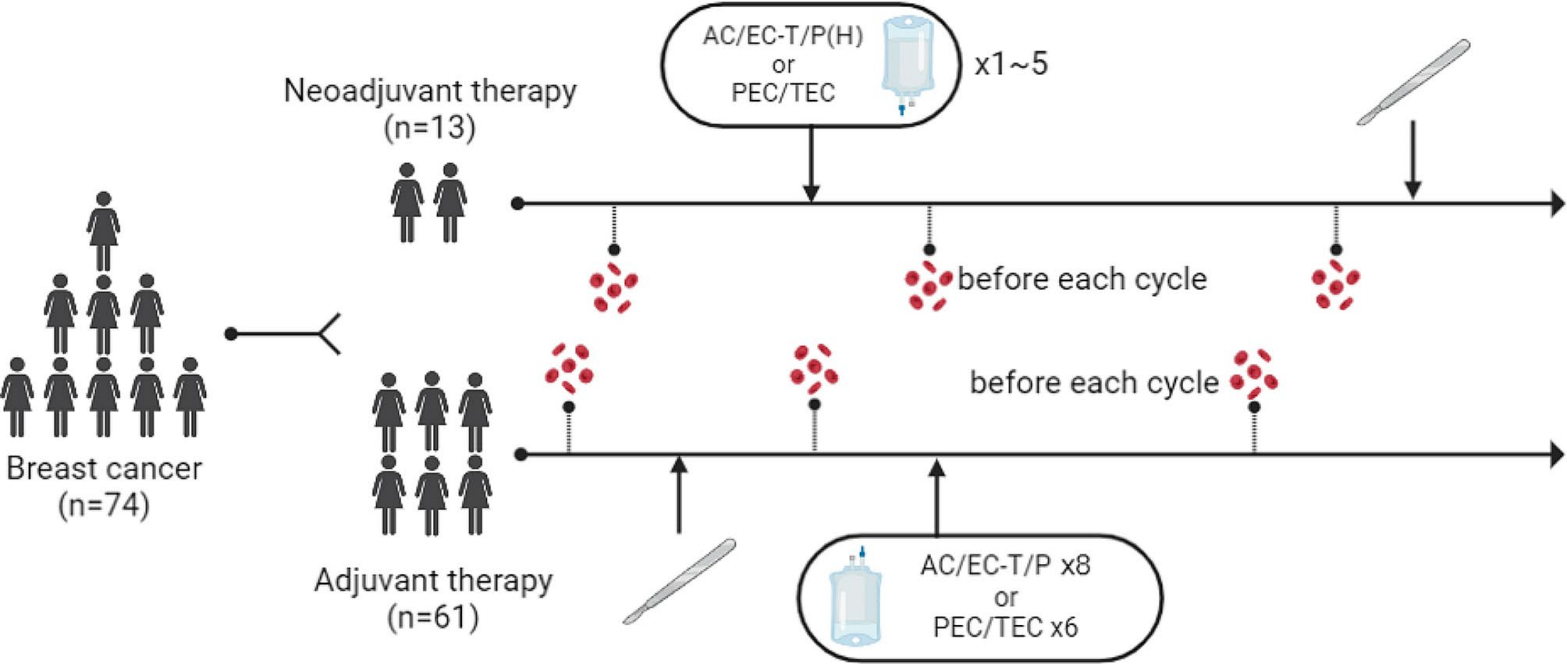
Sampling process of PB samples from 74 BC patients. The 74 breast cancer patients were divided into NAT group (*n* = 13) and Adjuvant therapy group (*n* = 61) according to treatment methods. Peripheral blood of the patients was detected by flow cytometry before treatment or surgery. Abbreviations: AC, adriamycin combined with cyclophosphamide; EC, epirubicin combined with cyclophosphamide; T, docetaxel; P, paclitaxel; H, herceptin; PEC, paclitaxel combined with epirubicin and cyclophosphamide; TEC, docetaxel combined with epirubicin and cyclophosphamide

The clinical characteristics of the patients are listed in Table 2. PB samples were collected from 13 patients who underwent NAT and 61 patients who received adjuvant chemotherapy, and blood was withdrawn before every treatment (Fig. 1). The follow-up period for patients was between 5 and 39 months (30.87 ± 7.80), and the follow-up time was April 2023. The primary end point was event-free survival (EFS), disease progression, recurrence, or death was considered an event.

**Table 2:** Clinical characteristics of 74 BC patients

Variables	Statistics (*N* = 74)
**Age at diagnosis, years**	
Median	50(42.25, 54.00)
≤ 50	40(54.05%)
> 50	34(45.95%)
**Histological grade**	
II	62(83.78%)
II-III	1(1.35%)
III	6(8.11%)
X	5(6.76%)
**BC Molecular Subtypes**	
HR + HER2-	35(47.30%)
HER2+	26(35.14%)
TN	13(17.57%)
**Lymph Node Metastasis**	
Positive	32(43.24%)
Negative	42(56.76%)
**Tumor Size (cm)**	
≤ 2	35(47.30%)
2–5	35(47.30%)
< 5	4(5.41%)

Abbreviations: HER2+, HER2-positive; HR + HER2-, hormone receptor-positive/HER2-negative; TNBC, triple-negative breast cancer

### Flow cytometry analysis of the immune cell population

PBL subset levels were determined by flow cytometry before each treatment cycle. EDTA-anticoagulated fresh whole blood samples were processed within 2 h of collection by erythrocyte lysis for 10 min using lysing solution (BD Biosciences). After red blood cell lysis, cells were washed with PBS and followed by staining with fluorochrome-conjugated antibodies for 20 min at room temperature. The stained cells were suspended and analyzed using flow cytometry.

Gating strategies of representative flow cytometry plots for PBLs is shown in Supplementary Fig. 1. The PBL subsets consisted of 12 items that were selected for detailed phenotypic analysis, as shown in Table 3. PBLs was analyzed using the following antibody combinations: CD3 + T cells (CD45 + CD3+), CD8 + T cells (CD45 + CD3 + CD8+), CD4 + T cells (CD45 + CD3 + CD4+), Treg cells (CD3 + CD4 + CD25+), PD-L1 + T cells (CD3 + CD274+), LAG-3 + T cells (CD3 + CD223+), TIM3 + T cells (CD3 + CD366+), CTLA-4 + T cells (CD3 + CD152+), CD19 + CD32 + B cells (CD19 + CD32+), NK cells (CD3-CD16 + CD56+). Flow cytometry antibodies were obtained from Biolegend, including CD45-PerCP (clone HI30), CD3- FITC (clone HIT3a), CD8-PE (clone SK1), CD4-APC (clone OKT4), CD25-PE (clone BC96), PD-1-PE (clone EH12.2H7), PD-L1-APC (clone 29E.2A3), TIM-3-PE (clone F38-2E2), Lag-3-APC (clone 7H2C65), CTLA-4-APC (clone BNI3), CD19-PE (clone HIB19), CD32-APC (clone FUN-2), CD56-APC (clone 5.1H11) (Supplementary Table 1).

**Table 3:** Markers, abbreviations and references of PBL subsets

	Marker	Abbreviation	Reference (%)
T cells	CD3	CD3 + T cells	67.00 ~ 76.00
cytotoxic T (Tc) cells	CD3/CD8	CD8 + T cells	21.00 ~ 32.00
T Helper (Th) cells	CD3/CD4	CD4 + T cells	35.00 ~ 46.00
Th/Tc cells	CD3/CD8/CD4	CD4+/CD8 + cells	1.09 ~ 2.17
regulatory T cells	CD3/CD4/CD25	Treg cells	7.48 ~ 15.09
PD-L1 + T cells	CD3/CD274	PD-L1 + T cells	92.35 ~ 98.18
PD-1 + T cells	CD3/CD279	PD-1 + T cells	15.96 ~ 26.32
LAG-3 + T cells	CD3/CD223	LAG-3 + T cells	1.21 ~ 3.03
TIM3 + T cells	CD3/CD366	TIM3 + T cells	0.90 ~ 2.06
CTLA-4 + T cells	CD3/CD152	CTLA-4 + T cells	11.06 ~ 28.84
B cells	CD19/CD32	CD19 + CD32 + B cells	10.36 ~ 16.87
naturel killer cells	CD3/CD16/CD56	NK cells	11.00 ~ 23.00

The reference values were established by the Second Xiangya Hospital of Central South University according to *the Guidelines for peripheral lymphocyte subsets by flow cytometry* issued by the Ministry of Health of the People’s Republic of China.

Flow cytometric analysis was conducted using a FACSCalibur flow cytometer (Becton-Dickinson, BD Biosciences).

### Statistical analysis

The data analysis was performed using SPSS (version 26.0), GraphPad Prism (version 8.0) and R Studio (version 2021.09.0 + 351) software. Normality and homogeneity of variance were assessed using the Shapiro-Wilk normality test and Levene’s test, respectively. If the data passed the normality test, parametric statistical tests (Independent Samples t-test, Paired-Samples t-test, and Welch t-test) or one-way ANOVA were performed. If the data did not pass the normality test, nonparametric statistical tests (Mann-Whitney U test and Kruskal-Wallis test) were used. The chi-squared test was employed to determine differences in proportions. Significance level was set at *p* < 0.05, with *** indicating *p* < 0.001, ** indicating *p* < 0.01, and * indicating *p* < 0.05.

## Results

### Characteristics of PBL subsets at baseline

**Fig. 2:**
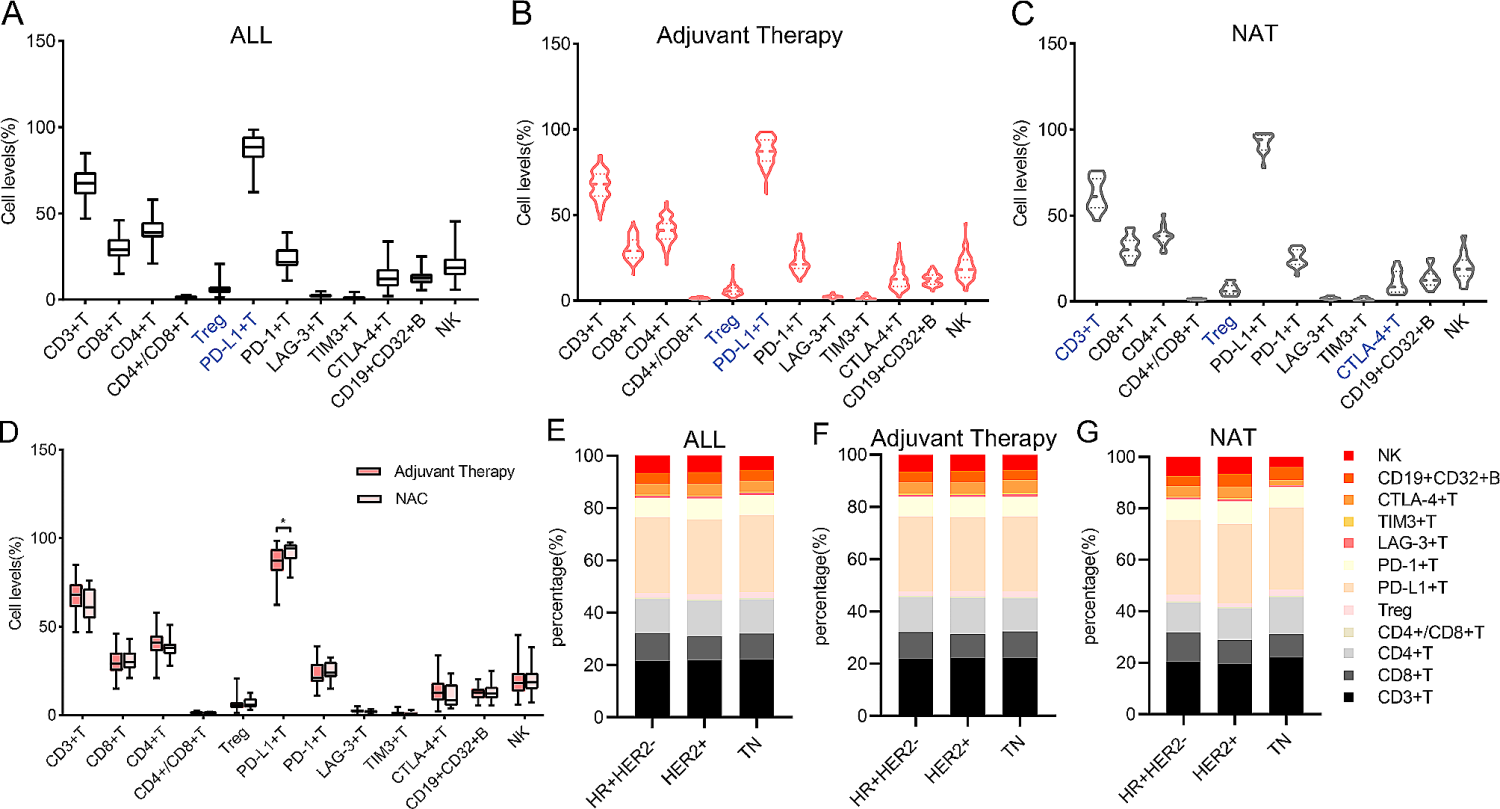
Features of PBL subsets before treatment. (**A**). Box plots of the distribution of baseline PBL subsets in all BC patients; blue text indicates below the reference value. (**B-C**). Baseline PBL subset characteristics of patients receiving (**B**) adjuvant therapy and (**C**) NAT. (**D**). Relationship between baseline PBL subsets and treatment regimen, Mann–Whitney U test. (**E-G**). The proportion of baseline PBL subsets in various molecular subtypes in (**E**) all patients and (**F**) patients receiving adjuvant therapy, (**G**) NAT. *P < 0.05, **P < 0.01, ***P < 0.001. Abbreviations: NAT, neoadjuvant therapy; HR + HER2-, hormone receptor-positive/HER2-negative; HER2+, HER2-positive; TN, triple-negative

To identify the characteristics of systemic immunity in patients with BC at baseline, we evaluated the levels of PBL subsets. The levels of Treg cells and PD-L1 + T cells in PB at baseline were lower than the reference value (Fig. 2A; Table 4). The patients who received adjuvant therapy had lower levels of Treg cells and PD-L1 + T cells than the reference values (Fig. 2B; Table 4). CD3 + T, Treg, and CTLA-4 + T cells were below reference values at baseline in patients on NAT (Fig. 2C; Table 4). We found that patients who received NAT had higher median pretreatment PD-L1 + T cell levels than patients who received adjuvant therapy (*p* = 0.028) (Fig. 2D).

**Table 4:** Median lymphocyte subset levels in different BC subtypes

**Lymphocyte subsets**	**Reference (%)**	**Baseline**		
		**ALL**	**Adjuvant therapy**	**Neoadjuvant therapy**
CD3 + T	67.00–76.00	67.50(61.00,73.50)	68.00(61.00,74.00)	61.00*(55.00,71.00)
CD8 + T	21.00–32.00	29.00(25.25,35.00)	29.00(25.00,35.00)	30.00(27.00,35.00)
CD4 + T	35.00–46.00	39.00(36.00,45.00)	41.00(36.00,45.00)	38.00(35.00,39.00)
CD4+/CD8 + T	1.09–2.17	1.33(1.04,1.78)	1.32(1.05,1.79)	1.33(1.01,1.48)
Treg	7.48–15.09	**5.82** ^*^ **(3.93,7.58)**	**5.51** ^*^ **(3.88,7.38)**	**5.85** ^*^ **(4.72,9.29)**
PD-L1 + T	92.35–98.18	**88.41** ^*^ **(82.22,94.58)**	**87.21** ^*^ **(81.60,93.77)**	94.14(88.64,96.61)
PD-1 + T	15.96–26.32	21.81(19.31,29.13)	21.17(19.00,28.90)	24.14(21.76,29.96)
LAG-3 + T	1.21–3.03	2.24(1.77,2.67)	2.30(1.78,2.67)	1.92(1.57,2.67)
TIM3 + T	0.90–2.06	1.05(0.67,1.91)	1.07(0.66,1.91)	0.96(0.75,1.81)
CTLA-4 + T	11.06–28.84	11.95(7.82,17.71)	12.46(8.28,17.77)	**8.50** ^*^ **(5.62,16.56)**
CD19 + CD32 + B	10.36–16.87	12.64(9.59,15.00)	12.88(9.53,14.81)	12.32(10.43,15.74)
NK	11.00–23.00	18.51(14.35,23.66)	18.13(13.47,23.83)	18.70(14.91,22.55)
**Lymphocyte subsets**	**Reference (%)**	**Baseline**		
		**HR + HER2-**	**HER2+**	**TNBC**
CD3 + T	67.00–76.00	69.00(59.00,72.00)	**65.00** ^*^ **(61.00,71.75)**	68.00(62.00,76.00)
CD8 + T	21.00–32.00	31.00(27.00,36.00)	28.00(24.25,30.00)	28.00(27.00,38.00)
CD4 + T	35.00–46.00	38.00(35.00,45.00)	41.50(37.25,44.75)	41.00(36.00,43.00)
CD4+/CD8 + T	1.09–2.17	1.25(0.96,1.64)	1.52(1.22,1.84)	1.48(0.93,1.67)
Treg	7.48–15.09	**4.97** ^*^ **(3.96,6.49)**	**5.15** ^*^ **(3.52,7.57)**	6.38*(5.84,7.99)
PD-L1 + T	92.35–98.18	**88.64** ^*^ **(85.04,93.63)**	**86.73** ^*^ **(81.38,93.74)**	93.42(88.04,96.96)
PD-1 + T	15.96–26.32	21.04(19.31,26.89)	24.01(21.13,29.49)	22.49(19.00,29.87)
LAG-3 + T	1.21–3.03	2.10(1.77,2.76)	2.08(1.79,2.50)	2.40(1.73,3.23)
TIM3 + T	0.90–2.06	1.08(0.75,1.88)	1.08(0.65,1.92)	**0.75** ^*^ **(0.54,1.83)**
CTLA-4 + T	11.06–28.84	12.46(8.25,17.28)	11.95(7.72,18.07)	11.77(7.77,16.55)
CD19 + CD32 + B	10.36–16.87	12.32(10.05,13.81)	13.55(9.21,16.33)	12.41(8.80,15.21)
NK	11.00–23.00	18.40(14.38,24.14)	18.01(15.24,21.73)	18.70(10.28,22.36)

In addition, Treg and PD-L1 + T cell levels at baseline were lower than the reference values in HR + HER2- subtypes. The baseline levels of CD3 + T, Treg, and PD-L1 + T cells were lower than the reference values in HER2 + subtypes. Treg and TIM3 + T cell levels were below the reference values in TN subtypes (Fig. 2E and G; Table 4). However, there were no significant differences among the three molecular subtypes in baseline PBL subsets (Supplementary Fig. 2).

### Alteration of PBL subsets in patients with BC after treatment

**Fig. 3:**
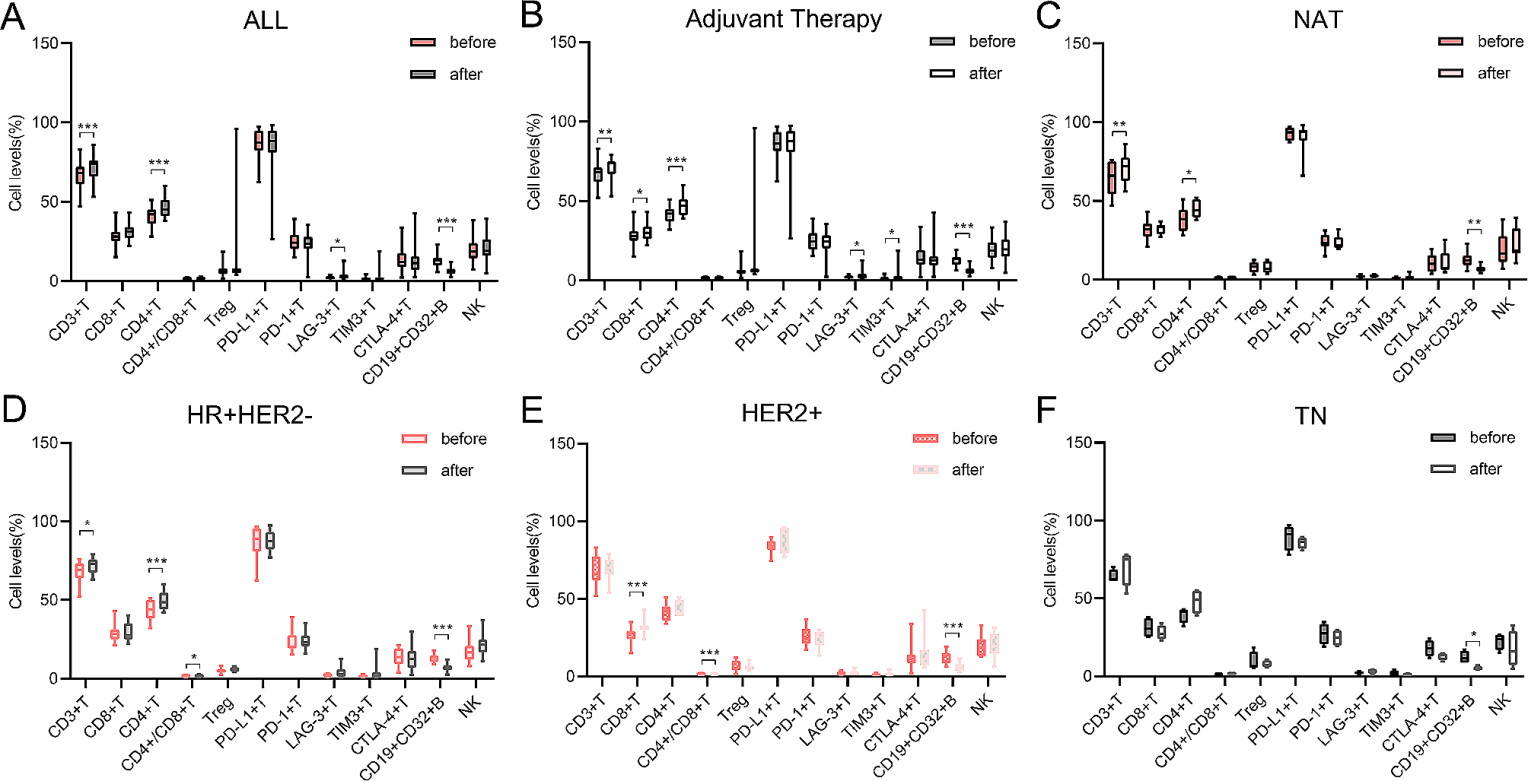
Variations in PBL subsets during systemic therapy. (**A-C**). PBL subsets before and after treatment were available for (**A**) all BC patients and patients receiving (**B**) adjuvant therapy and (**C**) NAT. (**D-E**). PBL subsets before and after adjuvant therapy were available for (**D**) HR + HER2-, (**E**) HER2 + and (**F**) TN. Paired T test. *P < 0.05, **P < 0.01, ***P < 0.001. Abbreviations: NAT, neoadjuvant therapy; HR + HER2-, hormone receptor-positive/HER2-negative; HER2+, HER2-positive; TN, triple-negative

Considering the effectiveness of treatment on the immune system in cancer patients [36–38], we explored the variations in PBL subsets before and after treatment. Compared with baseline levels, CD3 + T (*p* = 6.95e-05), CD4 + T (*p* = 9.22e-06) and LAG-3 + T (*p* = 0.016) cell levels were increased, while CD19 + CD32 + B (*p* = 5.89e-13) cell levels were decreased after treatment (Fig. 3A). After adjuvant therapy, CD3 + T (*p* = 0.0030), CD8 + T (*p* = 0.04), CD4 + T (*p* = 0.00020), LAG-3 + T (*p* = 0.030) and TIM-3 + T (*p* = 0.030) cell levels increased, whereas CD19 + CD32 + B (*p* = 1.99e-10) cell levels decreased (Fig. 3B). The levels of CD3 + T (*p* = 0.0041) and CD4 + T (*p* = 0.030) cells were significantly increased, while those of CD19 + CD32 + B (*p* = 0.0021) cells were significantly decreased after NAT (Fig. 3C).

Given the small number of patients receiving NAT, we focused the subtype analysis on patients who received adjuvant therapy. In the HR + HER2- subtype, CD3 + T (*p* = 0.010), CD4 + T (*p* = 0.0010) and CD4+/CD8 + T (*p* = 0.042) cells increased and CD19 + CD32 + B (*p* = 1.03e-05) cells decreased significantly after treatment (Fig. 3D). In HER2 + patients, CD8 + T (*p* = 8.84e-05) cells increased after treatment and CD4+/CD8 + T (*p* = 0.0094) cells and CD19 + CD32 + B cells (*p* = 0.0002) decreased after treatment (Fig. 3E). Among the TN subtypes, only CD19 + CD32 + B (*p* = 0.038) cells showed a significant decrease (Fig. 3F).

These results suggest that treatment leads to changes in the composition of PBL subsets in BC patients. The changes may be related to the molecular subtype.

### Changes in PBL subsets caused by chemotherapeutic drugs

**Fig. 4:**
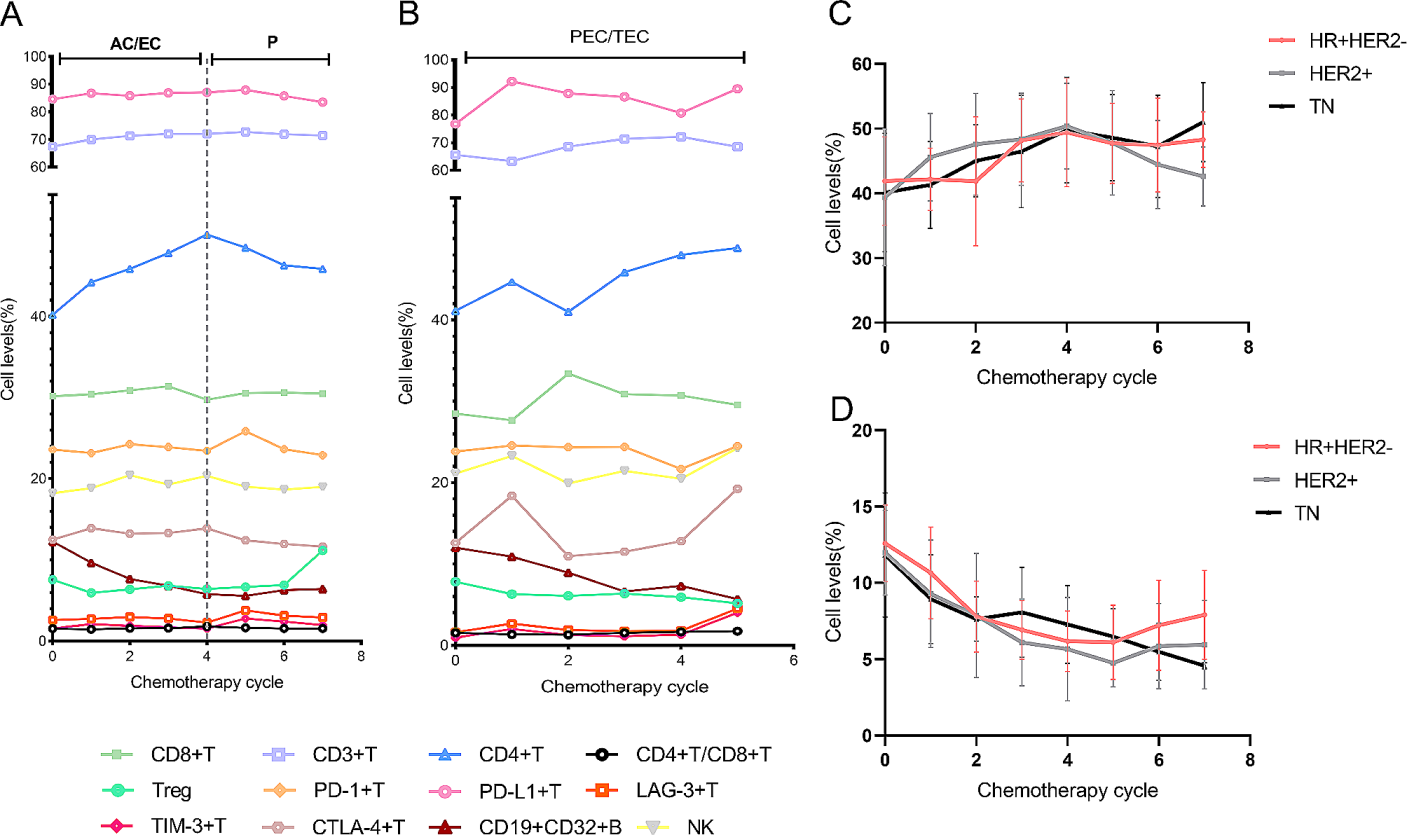
PBL subsets are vulnerable to chemotherapeutic drugs. (**A**). Changes in PBL subsets in patients treated with the AC/EC-T/P regimen. (**B**). Changes in PBL subsets in patients treated with the PEC/TEC regimen. (**C-D**). Changes in (**C**) CD4 + T and (**D**) CD19 + CD32 + B in different BC molecular subtypes during adjuvant therapy chemotherapy cycles. Abbreviations: HR + HER2-, hormone receptor-positive/HER2-negative; HER2+, HER2-positive; TN, triple-negative; AC, adriamycin combined with cyclophosphamide; EC, epirubicin combined with cyclophosphamide; T, docetaxel; P, paclitaxel; H, herceptin; PEC, paclitaxel combined with epirubicin and cyclophosphamide; TEC, docetaxel combined with epirubicin and cyclophosphamide

To further explore the specific reasons for the changes in PBL subsets caused by chemotherapy, we analysed the PBL subsets of patients before chemotherapy in each cycle. Interestingly, treatment with different chemotherapy regimens also contributed to the distinct lymphocyte variations. In patients treated with the AC/EC-T/P course, the levels of CD4 + T cells were time dependent, while PEC/TEC did not. Additionally, they increased during the first four cycles of AC/EC-T/P but decreased with the addition of paclitaxel drugs. The levels of CD19 + CD32 + B declined with chemotherapy cycles, regardless of the type of chemotherapeutic drugs (Fig. 4A, B). These findings indicate that paclitaxel seemingly has an inhibitory effect on CD4 + T cells. In patients with different molecular subtypes of BC who received adjuvant therapy, the trends of changes in both CD4 + T and CD19 + CD32 + B cells were basically the same (Fig. 4C, D). Altogether, our results suggest that the changes in CD4 + T cells induced by chemotherapy may be related to the types of drugs.

### Prognostic value of baseline PBL subsets in BC

**Fig. 5:**
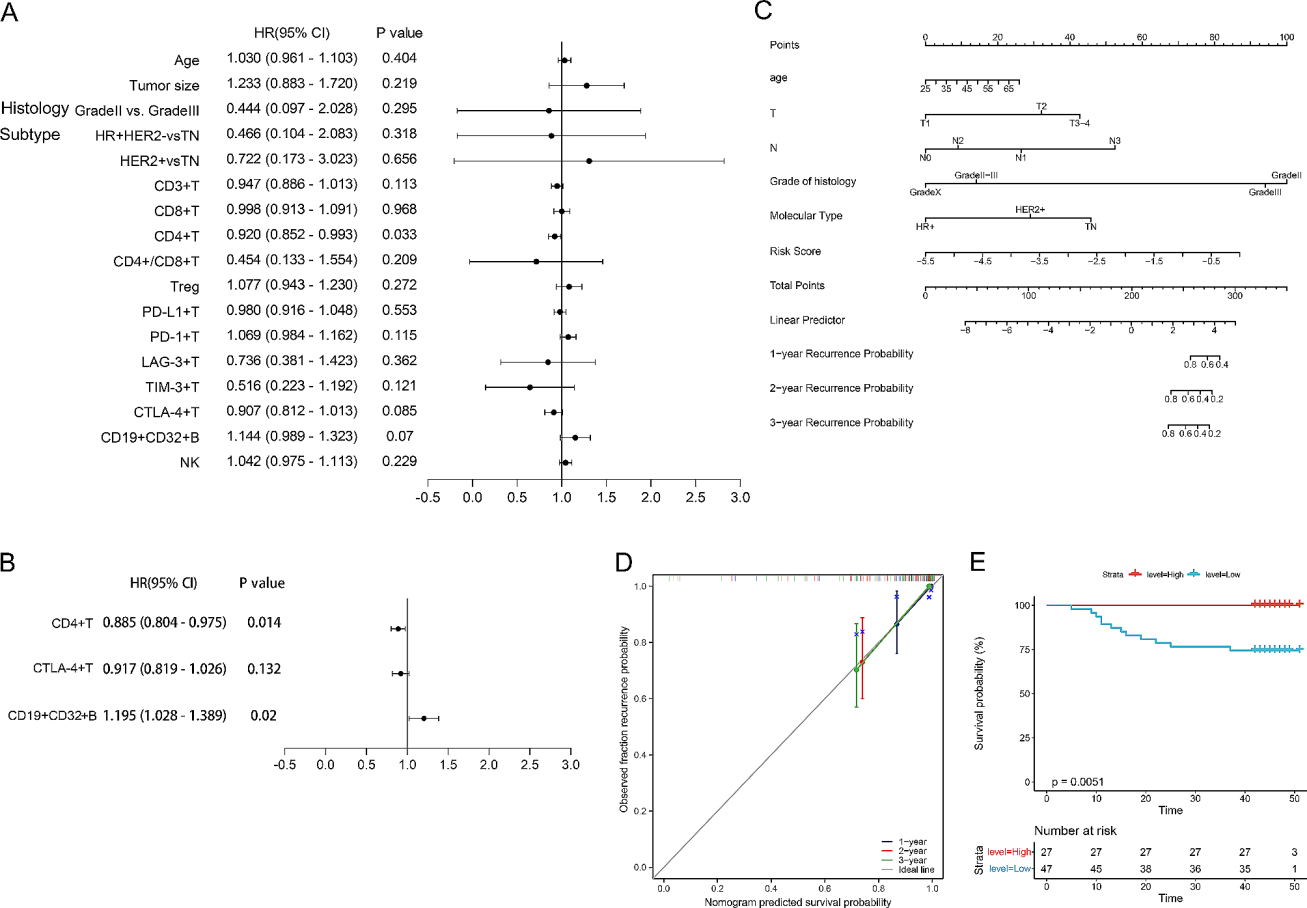
The clinical significance of the baseline PBL subsets. (**A**) Forest plot of independent predictors of prognosis in BC patients, univariate Cox regression analysis. (**B**) Forest plot of independent predictors of prognosis in BC patients, multivariate Cox regression analysis. (**C**) Nomogram to evaluate model performance. (**D**) Calibration curve to evaluate model performance. (**E**) KM EFS curve of CD4 + T cells in BC patients

We investigated the clinical significance of peripheral blood cell subsets at baseline in patients with BC. In univariate Cox regression analysis, CD4 + T cells (HR 0.92, 95% CI 0.852–0.993, *p* = 0.033) were found to be an important predictor of prognosis (Fig. 5A). The variables with *p*-values ≤ 0.10 from univariate Cox regression analysis were included in the multivariate Cox regression model, which incorporated CD4 + T cells, CTLA-4 + T cells, and CD19 + CD32 + B cells.

The results indicate that CD4 + T cells (HR 0.885, 95% CI 0.804–0.975, *p* = 0.014) and CD19 + CD32 + B cells (HR 1.195, 95% CI 1.028–1.389, *p* = 0.02) independently predict prognosis (Fig. 5B). A risk formula was developed based on the results of multivariate Cox regression analysis. The risk score was calculated as follows: levels of CD4 + T cells*(-0.12177) + levels of CD19 + CD32 + B cells*(0.1785). The prognostic nomogram and calibration curve demonstrate the model’s high efficacy (Fig. 5C, D).

The patients were divided into two groups based on their PBL expression level, using a cut-off point of 42.00 for CD4 + T cells. The results suggested that patients with a higher baseline level of CD4 + T cells exhibited improved prognosis (*p* = 0.0051) (Fig. 5E). In summary, our results demonstrate model’s strong predictive ability for prognosis, with PB CD4 + T cells serving as an independent prognostic factor for BC patients.

## Discussion

BC is a prominent cause of death among women globally and presents difficulties in achieving successful patient treatment [1]. Increasing evidence indicates that the immune profiles of the host may influence patient outcomes in BC [21, 39, 40]. The immune system is involved in normal breast development and has led to extensive research on the immune landscape of BC patients. Multiple studies have demonstrated that increasing immunogenicity and T-cell infiltration in the PB are potential strategies for the success of BC treatment [31, 41–43].

Multiple studies have demonstrated the significant role of PBLs in the clinical cure of cancer, especially in the therapeutic response to chemotherapy and improving the survival time in BC patients. Feng et al. expounded that peripheral baseline CD3 + T cells, CD8 + T cells, and NK cells independently predicted pathological complete response (pCR) in BC patients receiving NAT [31]. Li et al. proposed that peripheral CD4 + cells possess a stable ability to predict all clinical outcomes in patients with metastatic triple-negative breast cancer (mTNBC). Furthermore, their study highlighted a significant correlation between this clinical prognosis prediction and chemotherapy [42]. Therefore, this new knowledge may in turn open up opportunities for the stratification and prognosis prediction of BC patients by the quantitative evaluation of PBLs. Upon T-cell receptor (TCR) activation and cytokine stimulation, CD4 + T cells have the capacity to differentiate into various T-helper (Th) cell lineages. These lineages play a crucial role in immune response by releasing specific cytokines in response to different pathogenic insults [44, 45]. CD4 + Th1 cells secrete type I cytokines, specifically interferon-gamma (IFN-γ), leading to the activation of antigen-presenting cells. This activation subsequently stimulates a CD8 + T cell response. On the other hand, CD4 + Th2 cells secrete type II cytokines, including IL-4, IL-5, and IL-13, which contribute to the enhancement of humoral immune responses [46]. It is generally considered that Th1highTh2low is a good indicator of antitumour activity [46]. In our study, we observed a significant alteration in the proportion of CD4 + T cells in PB after chemotherapy. Thus, it is very important to further study the function of CD4 + T cells in BC patients.

Previously, chemotherapy was believed to exert solely immunosuppressive effects. However, recent studies have displayed different immunogenicity patterns in cancer patients undergoing chemotherapy. These patterns arise due to the cytotoxic effects of chemotherapy, which facilitate immunogenic cell death and consequently lead to the release of antigens and danger signals. Such released components contribute to the recruitment of antigen-presenting cells, promote the engulfment of dying cells, and facilitate dendritic cell (DC) maturation. These processes are vital for T-cell priming [33, 47]. Together, these signals stimulate and activate the immune system for antigen recognition and tumor cell elimination by recruiting and activating immune cells [48]. Several kinds of chemotherapy drugs, including anthracyclines, cyclophosphamide, and microtubule-stabilizing agents, are often used in BC. In our study, we found that different chemotherapy drugs may induce different responses to peripheral immunity. The cause of this phenomenon is unclear, but probably related to the mechanism of action of chemotherapy drugs.

In this study, we further established a nomogram containing PBLs to predict patient survival. Within lymphocyte subsets, higher baseline CD4 + and lower baseline CD19 + CD32 + B in PB correlated slightly with a better recurrence-free survival (RFS) in a multivariate Cox model. Previous studies on PBLs were limited to one subtype of breast cancer. A study involving 157 TNBC patients with MBC revealed that decreased pretreatment CD4 + cells in PB were strongly associated with a poorer prognosis [49]. Studies conducted by Jian Yang et al. and Xiao-Ran Liu et al. indicated that plasma CD4 + and peripheral cytotoxic T lymphocytes (pCTLs) were identified as independent negative predictors of progression-free survival (PFS) in HER2 + patients [50, 51]. However, there is no clear biological explanation as to why the same PBL subgroups may have inconsistent prognostic value in different BC subtypes. In our predictive model, we accounted for breast cancer subtypes as a variable to avoid the problem caused by tumor heterogeneity. The performance of a nomogram must be evaluated through calibration and discrimination. Internal validation in this study demonstrated strong discriminatory power (C-index: 0.852) with the inclusion of PBLs in the nomogram. Our nomogram was well calibrated to predict RFS. Therefore, our study can provide noninvasive, accurate and easily accessible predictors for survival in BC patients and assist clinicians in gaining a better understanding of tumor immunity. There are some limitations in this study. First, this is a retrospective study, and the follow-up time of patients involved in this research was short. The results should be further confirmed by prospective studies, and we will continue the follow-up in the long term. Second, the specific type of PBL subsets needs to be explored in depth.

## Conclusions

In summary, our results suggest that PBL subsets are helpful in elucidating the systemic immune response of BC. Additionally, the nomogram that incorporates PBLs demonstrated accurate predictions of individualized survival probabilities for BC patients. This practical model has the potential to provide support to clinicians and patients when making clinical decisions and optimizing treatment strategies.

### Electronic supplementary material

Below is the link to the electronic supplementary material.

Supplementary Material 1

Supplementary Material 2

## Data Availability

The data that support the findings of this study are available from the corresponding author upon reasonable request.

## Abbreviations

BC: Breast cancer
PBLs: Peripheral blood lymphocytes
NAT: Neoadjuvant therapy
EFS: Event-free survival
TNBC: Triple-negative breast cancer
ICIs: Immune checkpoint inhibitors
CAR-T: Chimeric antigen receptor T
TILs: Tumor infiltrating lymphocytes
OS: Overall survival
PB: Peripheral blood
HR + HER2-: ER or PR positive, or both HER2 negative
HER2+: HER2 positive
pCR: Pathological complete response
mTNBC: Metastatic triple-negative breast cancer
TCR: T-cell receptor
Th: T-helper
IFN-γ: Interferon-gammal
DC: Dendritic cell
RFS: Recurrence-free survival
pCTLs: Peripheral cytotoxic T lymphocytes
AC: Adriamycin combined with cyclophosphamide
EC: Epirubicin combined with cyclophosphamide
T: Docetaxel
P: Paclitaxel
H: Herceptin
PEC: Paclitaxel combined with epirubicin and cyclophosphamide
TEC: Docetaxel combined with epirubicin and cyclophosphamide

## References

[1] Siegel RL, Miller KD, Wagle NS, Jemal A (2023). Cancer statistics, 2023. CA Cancer J Clin.

[2] Sung H, Ferlay J, Siegel RL, Laversanne M, Soerjomataram I, Jemal A (2021). Global Cancer statistics 2020: GLOBOCAN estimates of incidence and Mortality Worldwide for 36 cancers in 185 countries. Cancer J Clin.

[3] Loibl S, Marme F, Martin M, Untch M, Bonnefoi H, Kim SB (2021). Palbociclib for residual high-risk invasive HR-Positive and HER2-Negative early breast Cancer-the Penelope-B Trial. J Clin Oncol.

[4] Emens LA (2018). Breast Cancer Immunotherapy: facts and hopes. Clin cancer Research: Official J Am Association Cancer Res.

[5] Cortes J, Cescon DW, Rugo HS, Nowecki Z, Im SA, Yusof MM (2020). Pembrolizumab plus chemotherapy versus placebo plus chemotherapy for previously untreated locally recurrent inoperable or metastatic triple-negative breast cancer (KEYNOTE-355): a randomised, placebo-controlled, double-blind, phase 3 clinical trial. Lancet (London England).

[6] Schmid P, Cortes J, Pusztai L, McArthur H, Kümmel S, Bergh J (2020). Pembrolizumab for early triple-negative breast Cancer. N Engl J Med.

[7] Schmid P, Adams S, Rugo HS, Schneeweiss A, Barrios CH, Iwata H (2018). Atezolizumab and Nab-Paclitaxel in Advanced Triple-negative breast Cancer. N Engl J Med.

[8] Keam SJ, Tremelimumab (2023). First Approval Drugs.

[9] Grubczak K, Kretowska-Grunwald A, Groth D, Poplawska I, Eljaszewicz A, Bolkun L et al. Differential Response of MDA-MB-231 and MCF-7 breast Cancer cells to in Vitro inhibition with CTLA-4 and PD-1 through Cancer-Immune cells modified interactions. Cells. 2021;10(8). 10.3390/cells10082044 PMC8392578 34440813

[10] Adams S, Othus M, Patel SP, Miller KD, Chugh R, Schuetze SM (2022). A Multicenter Phase II Trial of Ipilimumab and Nivolumab in Unresectable or metastatic metaplastic breast Cancer: Cohort 36 of dual Anti-CTLA-4 and Anti-PD-1 blockade in rare tumors (DART, SWOG S1609). Clin cancer Research: Official J Am Association Cancer Res.

[11] Corti C, Venetis K, Sajjadi E, Zattoni L, Curigliano G, Fusco N (2022). CAR-T cell therapy for triple-negative breast cancer and other solid tumors: preclinical and clinical progress. Expert Opin Investig Drugs.

[12] Tchou J, Zhao Y, Levine BL, Zhang PJ, Davis MM, Melenhorst JJ (2017). Safety and Efficacy of Intratumoral Injections of Chimeric Antigen Receptor (CAR) T cells in metastatic breast Cancer. Cancer Immunol Res.

[13] Miles D, Gligorov J, André F, Cameron D, Schneeweiss A, Barrios C (2021). Primary results from IMpassion131, a double-blind, placebo-controlled, randomised phase III trial of first-line paclitaxel with or without atezolizumab for unresectable locally advanced/metastatic triple-negative breast cancer. Annals of Oncology: Official Journal of the European Society for Medical Oncology.

[14] Hattori M, Masuda N, Takano T, Tsugawa K, Inoue K, Matsumoto K et al. Pembrolizumab plus chemotherapy in Japanese patients with triple-negative breast cancer: results from KEYNOTE-355. Cancer Med. 2023. 10.1002/cam4.5757 PMC10225213 36916728

[15] Loi S, Salgado R, Schmid P, Cortes J, Cescon DW, Winer EP (2023). Association between Biomarkers and Clinical Outcomes of Pembrolizumab Monotherapy in patients with metastatic triple-negative breast Cancer: KEYNOTE-086 exploratory analysis. JCO Precis Oncol.

[16] Rugo HS, Delord JP, Im SA, Ott PA, Piha-Paul SA, Bedard PL (2018). Safety and Antitumor Activity of Pembrolizumab in patients with estrogen Receptor-Positive/Human epidermal growth factor receptor 2-Negative advanced breast Cancer. Clin cancer Research: Official J Am Association Cancer Res.

[17] Nanda R, Liu MC, Yau C, Shatsky R, Pusztai L, Wallace A (2020). Effect of Pembrolizumab Plus Neoadjuvant Chemotherapy on Pathologic Complete response in women with early-stage breast Cancer: an analysis of the Ongoing phase 2 adaptively randomized I-SPY2 trial. JAMA Oncol.

[18] Ali HR, Chlon L, Pharoah PD, Markowetz F, Caldas C (2016). Patterns of Immune infiltration in breast Cancer and their clinical implications: a gene-expression-based retrospective study. PLoS Med.

[19] Lee KH, Kim EY, Yun JS, Park YL, Do SI, Chae SW (2018). The prognostic and predictive value of tumor-infiltrating lymphocytes and hematologic parameters in patients with breast cancer. BMC Cancer.

[20] Adams S, Gray RJ, Demaria S, Goldstein L, Perez EA, Shulman LN (2014). Prognostic value of tumor-infiltrating lymphocytes in triple-negative breast cancers from two phase III randomized adjuvant breast cancer trials: ECOG 2197 and ECOG 1199. J Clin Oncol.

[21] Stanton SE, Disis ML (2016). Clinical significance of tumor-infiltrating lymphocytes in breast cancer. J Immunother Cancer.

[22] Ahn SG, Jeong J, Hong S, Jung WH (2015). Current issues and clinical evidence in Tumor-infiltrating lymphocytes in breast Cancer. J Pathol Transl Med.

[23] Egelston CA, Avalos C, Tu TY, Rosario A, Wang R, Solomon S et al. Resident memory CD8 + T cells within cancer islands mediate survival in breast cancer patients. JCI Insight. 2019;4(19). 10.1172/jci.insight.130000 PMC6795408 31465302

[24] Lowenfeld L, Xu S, Czerniecki BJ (2019). CD4(+) Th1 to the rescue in HER-2 + breast cancer. Oncoimmunology.

[25] Noel G, Fontsa ML, Garaud S, De Silva P, de Wind A, Van den Eynden GG (2021). Functional Th1-oriented T follicular helper cells that infiltrate human breast cancer promote effective adaptive immunity. J Clin Invest.

[26] Zheng Z, Li YN, Jia S, Zhu M, Cao L, Tao M (2021). Lung mesenchymal stromal cells influenced by Th2 cytokines mobilize neutrophils and facilitate metastasis by producing complement C3. Nat Commun.

[27] Plitas G, Konopacki C, Wu K, Bos PD, Morrow M, Putintseva EV (2016). Regulatory T cells exhibit distinct features in human breast Cancer. Immunity.

[28] Thompson E, Taube JM, Elwood H, Sharma R, Meeker A, Warzecha HN (2016). The immune microenvironment of breast ductal carcinoma in situ. Mod Pathol.

[29] Zhou J, Lin HP, Xu X, Wang XH, Rong L, Zhang Y (2022). The predictive value of peripheral blood cells and lymphocyte subsets in oesophageal squamous cell cancer patients with neoadjuvant chemoradiotherapy. Front Immunol.

[30] Mao F, Yang C, Luo W, Wang Y, Xie J, Wang H (2022). Peripheral blood lymphocyte subsets are associated with the clinical outcomes of prostate cancer patients. Int Immunopharmacol.

[31] Feng J, Yi J, Zouxu X, Li J, Xiong Z, Huang X (2022). Peripheral blood lymphocytes subtypes as new predictors for neoadjuvant therapy efficacy in breast cancer. Cancer Med.

[32] Mittal D, Gubin MM, Schreiber RD, Smyth MJ (2014). New insights into cancer immunoediting and its three component phases–elimination, equilibrium and escape. Curr Opin Immunol.

[33] Garg AD, More S, Rufo N, Mece O, Sassano ML, Agostinis P (2017). Trial watch: immunogenic cell death induction by anticancer chemotherapeutics. Oncoimmunology.

[34] Adams S, Gatti-Mays ME, Kalinsky K, Korde LA, Sharon E, Amiri-Kordestani L (2019). Current Landscape of Immunotherapy in breast Cancer: a review. JAMA Oncol.

[35] Harbeck N, Gnant M (2017). Breast cancer. Lancet (London England).

[36] Vanguri RS, Fenn KM, Kearney MR, Wang Q, Guo H, Marks DK (2022). Tumor Immune Microenvironment and Response to Neoadjuvant Chemotherapy in hormone Receptor/HER2 + early stage breast Cancer. Clin Breast Cancer.

[37] Waks AG, Stover DG, Guerriero JL, Dillon D, Barry WT, Gjini E (2019). The Immune Microenvironment in hormone receptor-positive breast Cancer before and after preoperative chemotherapy. Clin Cancer Res.

[38] Hodge JW, Garnett CT, Farsaci B, Palena C, Tsang KY, Ferrone S (2013). Chemotherapy-induced immunogenic modulation of tumor cells enhances killing by cytotoxic T lymphocytes and is distinct from immunogenic cell death. Int J Cancer.

[39] Garcia-Martinez E, Gil GL, Benito AC, Gonzalez-Billalabeitia E, Conesa MA, Garcia Garcia T (2014). Tumor-infiltrating immune cell profiles and their change after neoadjuvant chemotherapy predict response and prognosis of breast cancer. Breast Cancer Res.

[40] Denkert C, Loibl S, Noske A, Roller M, Muller BM, Komor M (2010). Tumor-associated lymphocytes as an independent predictor of response to neoadjuvant chemotherapy in breast cancer. J Clin Oncol.

[41] Jørgensen N, Lænkholm A-V, Sækmose SG, Hansen LB, Hviid TVF (2021). Peripheral blood immune markers in breast cancer: differences in regulatory T cell abundance are related to clinical parameters. Clin Immunol.

[42] Li M, Xu J, Jiang C, Zhang J, Sun T (2022). Predictive and prognostic role of Peripheral blood T-Cell subsets in Triple-negative breast Cancer. Front Oncol.

[43] Liu A, Xia Y, Li W, Zhang G, Liu Y, Ye S (2022). The predictive value of changes in the Absolute counts of Peripheral lymphocyte subsets for progression and prognosis in breast Cancer patients. Contrast Media Mol Imaging.

[44] Banuelos J, Lu NZ (2016). A gradient of glucocorticoid sensitivity among helper T cell cytokines. Cytokine Growth Factor Rev.

[45] Hsu CY, Fu SH, Chien MW, Liu YW, Chen SJ, Sytwu HK. Post-translational modifications of transcription factors harnessing the etiology and pathophysiology in Colonic diseases. Int J Mol Sci. 2020;21(9). 10.3390/ijms21093207 PMC7246881 32369982

[46] Gajewski TF, Schreiber H, Fu YX (2013). Innate and adaptive immune cells in the tumor microenvironment. Nat Immunol.

[47] Galluzzi L, Buqué A, Kepp O, Zitvogel L, Kroemer G (2017). Immunogenic cell death in cancer and infectious disease. Nat Rev Immunol.

[48] Kroemer G, Galluzzi L, Kepp O, Zitvogel L (2013). Immunogenic cell death in cancer therapy. Annu Rev Immunol.

[49] Trédan O, Manuel M, Clapisson G, Bachelot T, Chabaud S, Bardin-dit-Courageot C et al. Patients with metastatic breast cancer leading to CD4 + T cell lymphopaenia have poor outcome. European journal of cancer (Oxford, England: 1990). 2013;49(7):1673-82. 10.1016/j.ejca.2012.11.028 23265706

[50] Liu XR, Yu JJ, Song GH, Di LJ, Jiang HF, Yan Y (2021). Peripheral cytotoxic T lymphocyte predicts first-line progression free survival in HER2-positive advanced breast cancer. Breast (Edinburgh Scotland).

[51] Yang J, Xu J, E Y, Sun T (2019). Predictive and prognostic value of circulating blood lymphocyte subsets in metastatic breast cancer. Cancer Med.

