# Supplementary material for: Immune characteristics and clinical significance of peripheral blood lymphocytes in breast cancer
Source: BMC Cancer. 2024 Jan 9;24:50. doi: 10.1186/s12885-024-11815-8 (PMC10775541; doi:10.1186/s12885-024-11815-8)
Supplement: Supplementary file 1 — Supplementary Material 1 [file 12885_2024_11815_MOESM1_ESM.pdf]

Supplementary Figure

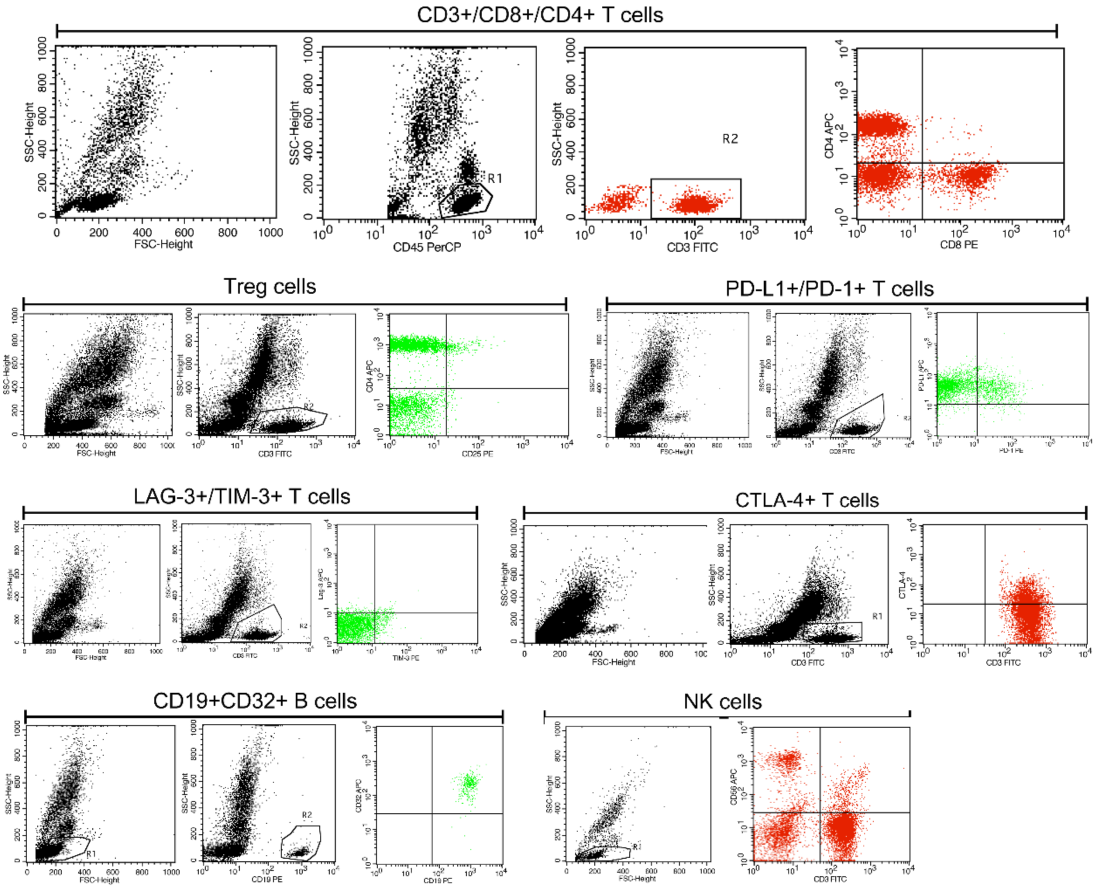

Supplementary Figure 1. Identification of representative gating strategies for PBL subsets. An overview of gating strategies for flow cytometry experiments.

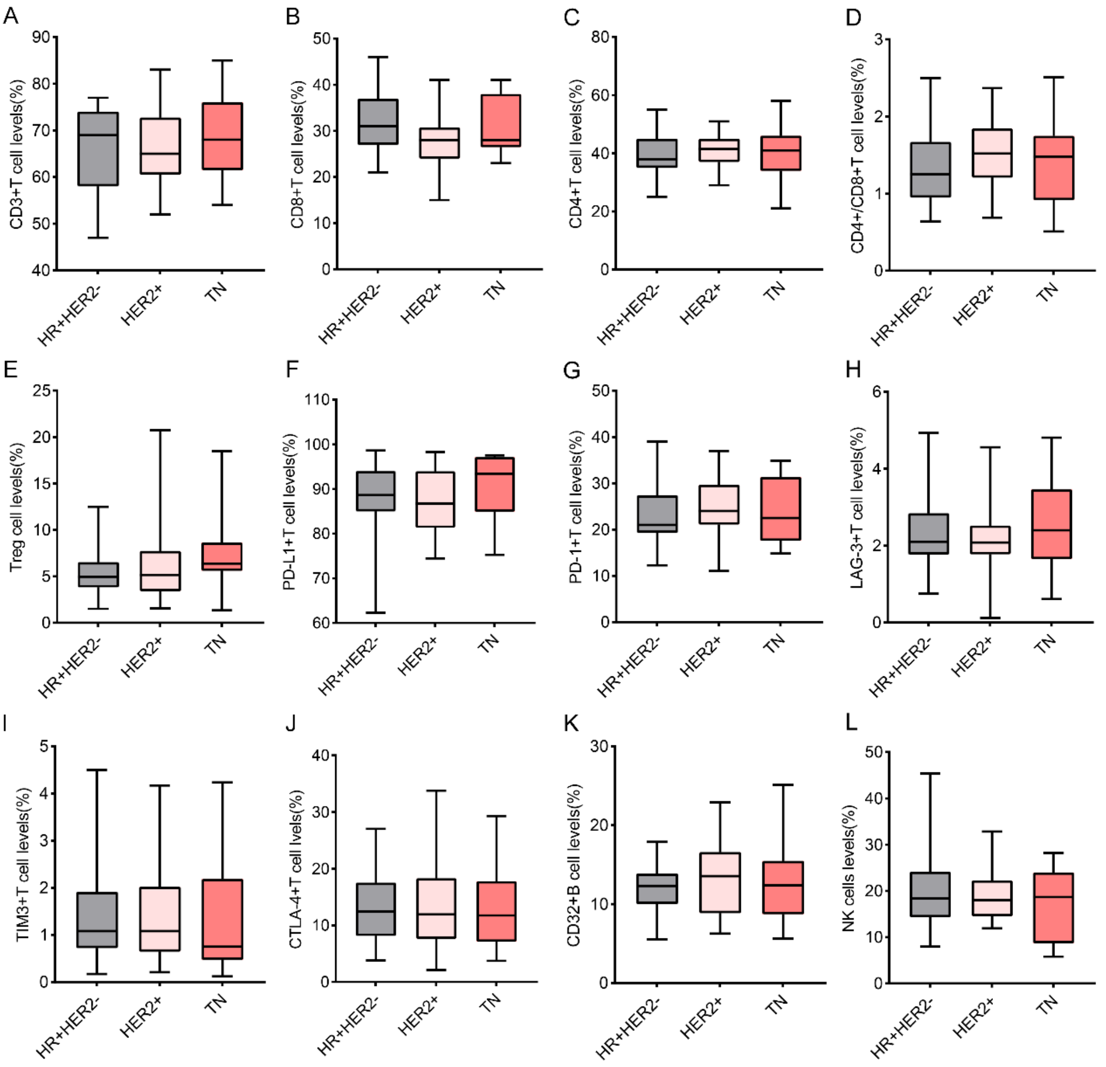

Supplementary Figure 2. The Box plot of baseline PBLs levels in different molecular subtypes.

(A)-(L) Levels of CD3+T (A), CD8+T(B), CD4+T(C), CD4+/CD8+T(D), Treg(E), PD-L1+T(F), PD-1+T(G), LAG-3+T(H), TIM3+T(I), CTLA-4+T(J), CD32+B(K), NK(L) in three molecular subtypes (HR+HER2-, HER2+, TN) of BC. Abbreviations: HR+HER2-, hormone receptor-positive/ HER2-negtive; HER2+, HER2-positive; TN, triple-negative; BC, breast cancer.
